# Datasets on the assessment of the scientific publication's corpora in circular economy and bioenergy approached from education and communication

**DOI:** 10.1016/j.dib.2023.108958

**Published:** 2023-02-08

**Authors:** Alejandro Carbonell-Alcocer, Juan Romero-Luis, Manuel Gertrudix, Daniel Wuebben

**Affiliations:** a Department of Audiovisual Communication and Advertising. Rey Juan Carlos University. Cam. del Molino, 5, 28942 Fuenlabrada, Madrid, Spain; b Arts and Humanities Division, IE University. C. Cardenal Zúñiga, 12, 40003, Segovia, Spain

**Keywords:** Systematic literature review (SLR), DESLOCIS framework, PRISMA statement, PRISMA Checklist, Cluster analysis

## Abstract

This article presents three datasets that specifically depict scientific literature published from 2009 to 2019 and that represent the overlaps between circular economy, bioenergy, education, and communication. All datasets have been obtained through an exhaustive methodological process based on a Systematic Literature Review (SLR). To collect data, we determined 12 Boolean Operators with words related to circular economy, bioenergy, communication, and education. Then, using the Publish or Perish software, 36 queries were made in the Web of Science, Scopus, and Google Scholar databases. Once the articles were retrieved, the Preferred Reporting Items for Systematic Reviews and Meta-Analyses (PRISMA) mode and PRISMA checklist were applied. 74 articles were then manually selected depending on their relationship with the field. Using the DESLOCIS framework, a wide evaluation of the articles was carried out focusing on the design, data collection, and analysis techniques. Thus, the first data set contains the metadata and metrics of the publications. The second data set details the analytical framework used. The third includes the analysis of the publication's corpora. Together, the data presents opportunities for longitudinal studies and meta-reviews in circular economy and bioenergy areas approached from perspectives of education and communication.

**Specifications Table**

**Table:** 

Subject	Social Sciences; Social science; Education; Communication
Specific subject area	Circular Economy; Bioenergy; Sustainability and the Environment.
Type of data	Tables Graphs
How the data were acquired	A common vocabulary was established. Circular economy and bioenergy experts from the BIOTRES-CM project (S2018/EMT-433) select five key terms. Based on an automated extraction process, the most repeated keywords in the field of communication and education in the last 10 years indexed in the Scimago Journal & Country Rank and the Journal Citation Reports were identified. Based on the selected words, an equation was generated using the logical operators AND and OR. Using Publish or Perish software [1], Boolean operators were entered in the title field to retrieve scientific publications from Web of Science, Scopus, and Google Scholar databases from 2009 to 2019. Applying the Preferred Reporting Items for Systematic Reviews and Meta-Analyses (PRISMA) statement and PRISMA checklist [2,3], articles, proceedings papers, and conference papers were selected. Once the final sample was established, the publications were studied employing a qualitative clustering process following the DESLOCIS framework (Descriptors for a systematic literature review on social sciences).
Data format	Raw Analyzed Filtered
Description of data collection	The selection of articles was carried out rigorously following the phases of identification, screening, and eligibility that are included in the PRISMA statement.
Data source location	Institution: Rey Juan Carlos University City/Town/Region: Fuenlabrada, Madrid Country: Spain Databases: Web of Science, Scopus, Google Scholar Years: 2009 to 2019
Data accessibility	[4]**Systematic Literature Review results: PRISMA Statement Phases for Education and Communication based on Circular Economy and Bioenergy Literature** Repository name: Zenodo. OpenAIRE. Direct URL to data: https://doi.org/10.5281/zenodo.4432436 [5]**Descriptors for a systematic literature review on social sciences (DESLOCIS)** Repository name: Zenodo. OpenAIRE. Direct URL to data: https://doi.org/10.5281/zenodo.4462764 [6]**Sample Records. A systematic review in Circular Economy and Bioenergy addressed by Education and Communication** Repository name: Zenodo. OpenAIRE. Direct URL to data: https://doi.org/10.5281/zenodo.4472402
Related research article	[7] Carbonell-Alcocer, A.; Romero-Luis, J.; Gertrudix, M. A Methodological Assessment Based on a Systematic Review of Circular Economy and Bioenergy Addressed by Education and Communication. Sustainability 2021, 13, 4273. https://doi.org/10.3390/su13084273 [8] Romero-Luis, J., Carbonell-Alcocer, A., Gertrudix, M., & Casado, M. D. C. G. (2021). What is the maturity level of circular economy and bioenergy research addressed from education and communication? A systematic literature review and epistemological perspectives. Journal of Cleaner Production, 322, 129007. https://doi.org/10.1016/j.jclepro.2021.129007

## Value of the Data

• The data collected allows for the identification of recent scientific publications and conference papers on circular economy and bioenergy from the perspectives of communication and education.
• The data provide insights into the impact of the current scientific literature including metadata and citations in Web of Science, Scopus, and Google Scholar databases.
• The DESLOCIS model (Descriptors for a systematic literature review on social sciences) offers a framework for the assessment of scientific literature related to the specific characteristics of publications in the social sciences.
• The data delves into the research corpora focusing on the collection techniques, the typology of the data, the characteristics of the samples, and the analysis techniques applied in the literature.
• The data provided may be useful for the development of longitudinal studies, meta-analyses, and comprehensive systematic literature reviews (SLR) as well as for carrying out replication studies.

## 1 Objective

Systematic literature reviews (SLR) facilitate the evaluation of the current state of the art of a specific field of knowledge. To ensure the replicability and reliability of the method, it is essential to use a standardized and systematic process. For this reason, the Preferred Reporting Items for Systematic Reviews and Meta-Analyses (PRISMA) Statement and the PRISMA Checklist have been applied. This protocol allows the scientific literature review process to be documented accurately and transparently.

The aim of the datasets is to provide a broad overview and snapshot of the state of the art based on the scientific articles and conference publications in the fields of circular economy and bioenergy addressed from the perspectives of education and communication.

## 2 Data Description

**Fig. 1:**
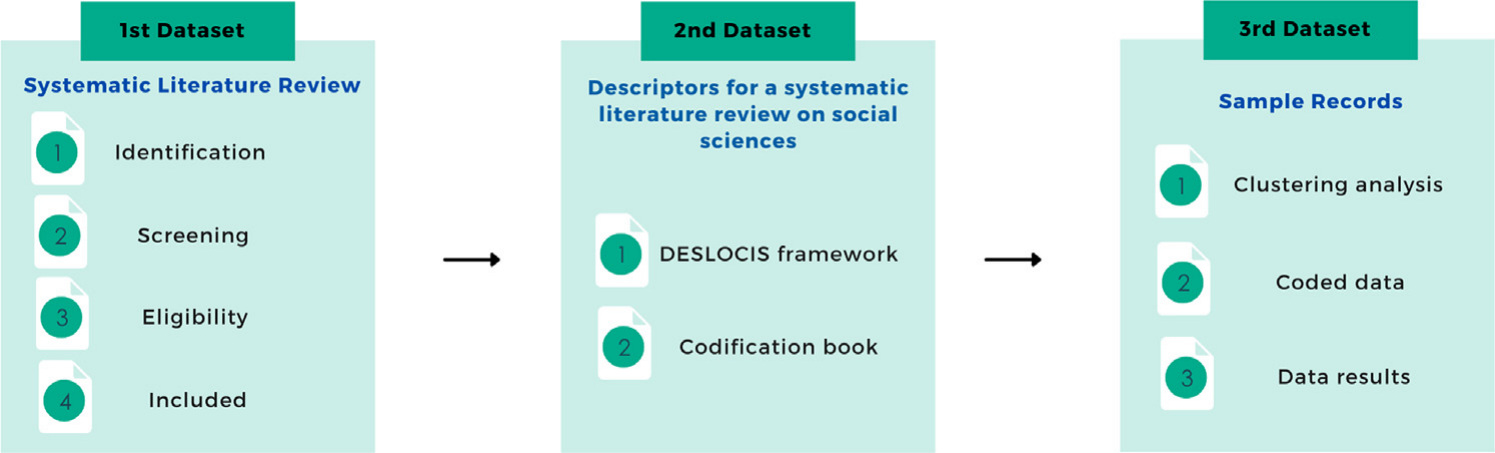
Datasets included in this data article and its corresponding files.

This data article is composed of 3 datasets. Fig. 1 depicts the content of each dataset and its files.

### 2.1 1st Data set: Systematic Literature Review results: PRISMA Statement Phases for Education and Communication based on Circular Economy and Bioenergy Literature

This repository includes four files related to the phases of the Systematic Literature Review (SLR) following the Preferred Reporting Items for Systematic Reviews and Meta-Analyses (PRISMA) statement: identification, screening, eligibility and included.

The first file contains, in separate excel sheets, the records identified through the search in Web of Science, Scopus, and Google Scholar depending on the Boolean operator included in Publish or Perish (Communication and Education). Table 1 contains the name of the columns and a description of the attribute queried.

**Table 1:** Column identification.

Column	Description
Title	Title of the article
Cites	Number of citations
Authors	Authors name
Year	Year of publication
Source	Journal/Conference/Proceeding paper
Publisher	Publisher name
ArticleURL	URL
CitesURL	Documents that have cited the paper
GSRank	Result ranking
Type	Type of publication (Article, editorial material, proceeding paper, review)
DOI	Digital Object Identifier
ISSN	Journal's ISSN
CitationURL	URL
Volume	Journal
Issue	Issue
StartPage	Start page
EndPage	End page
ECC	Estimated Citation Count
CitesPerYear	Yearly cites
CitesPerAuthor	Citation by author
AuthorCount	Number of authors
Age	Years after publications

The second file contains all the results screened merged in one excel sheet (n=259). It contains the following information: title, author, year, journal/conference, age, DOI, cites, GSRank, CitesPerYear, ECC, and author count. The values related to metric data from three databases have been included in separate columns distinguished by color.

The third file includes the eligible (n=170) and excluded (n=96) results as the outcome of the PRISMA statement phases. It contains the same columns as the second file. The publications have been eligible after manually evaluating their abstract to check whether they are related to the research topic.

The fourth file contains the final selection of publications (n=74). This data set was used to carry out the quantitative and qualitative synthesis.

### 2.2 2nd Data set: Descriptors for a systematic literature review on social sciences (DESLOCIS)

This repository includes an explanation of the methodological framework used for the analysis of the publications.

The objective of this model is to help researchers identify the design, data collection, and analysis of scientific publications in order to differentiate perspectives, approaches, and theories in existing and emerging research areas.

The repository contains the analysis variables and codification book. DESLOCIS framework is focused on the following variables: type of perspective, the scope of the object of study, field of knowledge, type of research, classification of objectives, declaration of objectives, declaration of hypothesis, declaration of study universe, sampling techniques, sample size, information collection techniques, dominant information collection technique, secondary information collection technique, source of data, nature of data, data collection, level of theoretical formalization, branch of knowledge, number of analysis techniques, predominant analysis technique and secondary analysis technique.

### 2.3 3rd Data set: Sample Records. A systematic review in Circular Economy and Bioenergy addressed by Education and Communication

The third repository incorporates three files related to the analytical procedure.

The first and second document contains the analyzed articles coded according to the DESLOCIS framework. There are two columns with the information of the article (Title and DOI) and 30 columns with the variables of the DESLOCIS framework.

The third document contains four excel sheets. The data in the sheets show univariable and multivariable statistical studies, frequencies and vertical percentages analysis, and the table of checks proportions by column using the Z test. It also includes stacked bar graphs.

## 3 Experimental Design, Materials and Methods

**Fig. 2:**
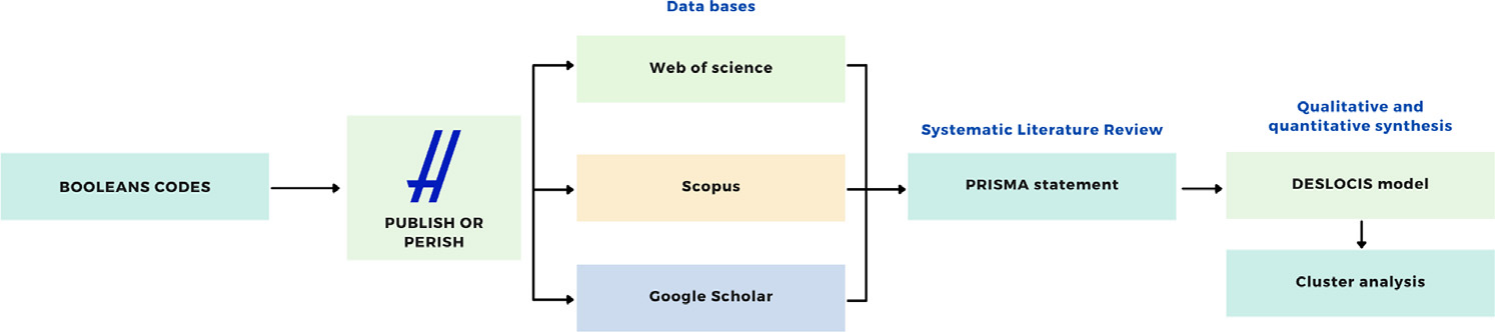
Process overview.

Given that the objective was to assess the state of the art, a rigorous methodological procedure has been followed. Fig. 2 depicts a general overview of the experimental design carried out.

A set of Boolean Operators was defined to recover the publications related to circular economy and bioenergy. For that reason, an equation combining general words related to circular economy and bioenergy with specific words linked to communication and education was used.

“General word” AND (“Specific word 1” OR “Specific word 2” OR “Specific word 3” […]).

Table 2 specifies the Boolean operators used to perform the searches in Web of Science, Scopus, and Google Scholar in the field of title from 2009 to 2019.

**Table 2:** Boolean operators.

Category	Boolean Operator
Communication	“urban biowastes” AND (“advertising” OR “communication” OR “communications” OR “media” OR “digital” OR “marketing” OR “effectiveness” OR “engagement” OR “education” OR “higher education” OR “persuasion” OR “memory” OR “credibility” OR “attention” OR “motivation” OR “ux” OR “user experience” OR “experience” OR “eye-tracking” OR “eye tracking” OR “usability” OR “digital content” OR “cross media” OR “transmedia” OR “360° video”)
Communication	“circular economy” AND (“advertising” OR “communication” OR “communications” OR “media” OR “digital” OR “marketing” OR “effectiveness” OR “engagement” OR “education” OR “higher education” OR “persuasion” OR “memory” OR “credibility” OR “attention” OR “motivation” OR “ux” OR “user experience” OR “experience” OR “eye-tracking” OR “eye tracking” OR “usability” OR “digital content” OR “cross media” OR “transmedia” OR “360° video”)
Communication	“chemical biobased products” AND (“advertising” OR “communication” OR “communications” OR “media” OR “digital” OR “marketing” OR “effectiveness” OR “engagement” OR “education” OR “higher education” OR “persuasion” OR “memory” OR “credibility” OR “attention” OR “motivation” OR “ux” OR “user experience” OR “experience” OR “eye-tracking” OR “eye tracking” OR “usability” OR “digital content” OR “cross media” OR “transmedia” OR “360° video”)
Communication	“bioenergy” AND (“advertising” OR “communication” OR “communications” OR “media” OR “digital” OR “marketing” OR “effectiveness” OR “engagement” OR “education” OR “higher education” OR “persuasion” OR “memory” OR “credibility” OR “attention” OR “motivation” OR “ux” OR “user experience” OR “experience” OR “eye-tracking” OR “eye tracking” OR “usability” OR “digital content” OR “cross media” OR “transmedia” OR “360° video”)
Communication	“biorefinery” AND (“advertising” OR “communication” OR “communications” OR “media” OR “digital” OR “marketing” OR “effectiveness” OR “engagement” OR “education” OR “higher education” OR “persuasion” OR “memory” OR “credibility” OR “attention” OR “motivation” OR “ux” OR “user experience” OR “experience” OR “eye-tracking” OR “eye tracking” OR “usability” OR “digital content” OR “cross media” OR “transmedia” OR “360° video”)
Communication	“urban biowaste” AND (“advertising” OR “communication” OR “communications” OR “media” OR “digital” OR “marketing” OR “effectiveness” OR “engagement” OR “education” OR “higher education” OR “persuasion” OR “memory” OR “credibility” OR “attention” OR “motivation” OR “ux” OR “user experience” OR “experience” OR “eye-tracking” OR “eye tracking” OR “usability” OR “digital content” OR “cross media” OR “transmedia” OR “360° video”)
Education	“urban biowastes” AND (“children” OR “education” OR “communication” OR “media literacy” OR “disinformation” OR “distance-learning” OR “e-learning” OR “information technology” OR “Information Communication Technology” OR “internet” OR “learning” OR “research” OR “school” OR “media” OR “social media” OR “social network” OR “student” OR “college students” OR “communication education” OR “learning experiment”)
Education	“circular economy” AND (“children” OR “education” OR “communication” OR “media literacy” OR “disinformation” OR “distance-learning” OR “e-learning” OR “information technology” OR “Information Communication Technology” OR “internet” OR “learning” OR “research” OR “school” OR “media” OR “social media” OR “social network” OR “student” OR “college students” OR “communication education” OR “learning experiment”)
Education	“chemical biobased products” AND (“children” OR “education” OR “communication” OR “media literacy” OR “disinformation” OR “distance-learning” OR “e-learning” OR “information technology” OR “Information Communication Technology” OR “internet” OR “learning” OR “research” OR “school” OR “media” OR “social media” OR “social network” OR “student” OR “college students” OR “communication education” OR “learning experiment”)
Education	“bioenergy” AND (“children” OR “education” OR “communication” OR “media literacy” OR “disinformation” OR “distance-learning” OR “e-learning” OR “information technology” OR “Information Communication Technology” OR “internet” OR “learning” OR “research” OR “school” OR “media” OR “social media” OR “social network” OR “student” OR “college students” OR “communication education” OR “learning experiment”)
Education	“biorefinery” AND (“children” OR “education” OR “communication” OR “media literacy” OR “disinformation” OR “distance-learning” OR “e-learning” OR “information technology” OR “Information Communication Technology” OR “internet” OR “learning” OR “research” OR “school” OR “media” OR “social media” OR “social network” OR “student” OR “college students” OR “communication education” OR “learning experiment”)
Education	“urban biowaste” AND (“children” OR “education” OR “communication” OR “media literacy” OR “disinformation” OR “distance-learning” OR “e-learning” OR “information technology” OR “Information Communication Technology” OR “internet” OR “learning” OR “research” OR “school” OR “media” OR “social media” OR “social network” OR “student” OR “college students” OR “communication education” OR “learning experiment”)

Then, to recover the publications, bibliographic software was used. Publish or Perish [1] is a specialized software that retrieves and analyzes academic publications. It recovers the metadata related to the publication and its citation metrics.

In order to conduct a Systematic Literature Review, it is necessary to establish the databases from which to retrieve the information, determine the time frame of the searches, the search field and the type of publication to be selected (scientific article, book, conference paper, reviews). A time frame of 10 years (2009 to 2019) was established, the title was set as a search field, and Web of Science, Scopus, and Google Scholar were defined as the main databases.

The 12 Boolean operators are introduced, and three databases were searched by executing 36 queries. Table 3 shows the results of the searches.

**Table 3:** Queries results.

	Web of Science	Scopus	Google Scholar	Total
Communication booleans	61	96	161	321
Education booleans	48	83	99	230
Total	109	179	263	551

**Fig. 3:**
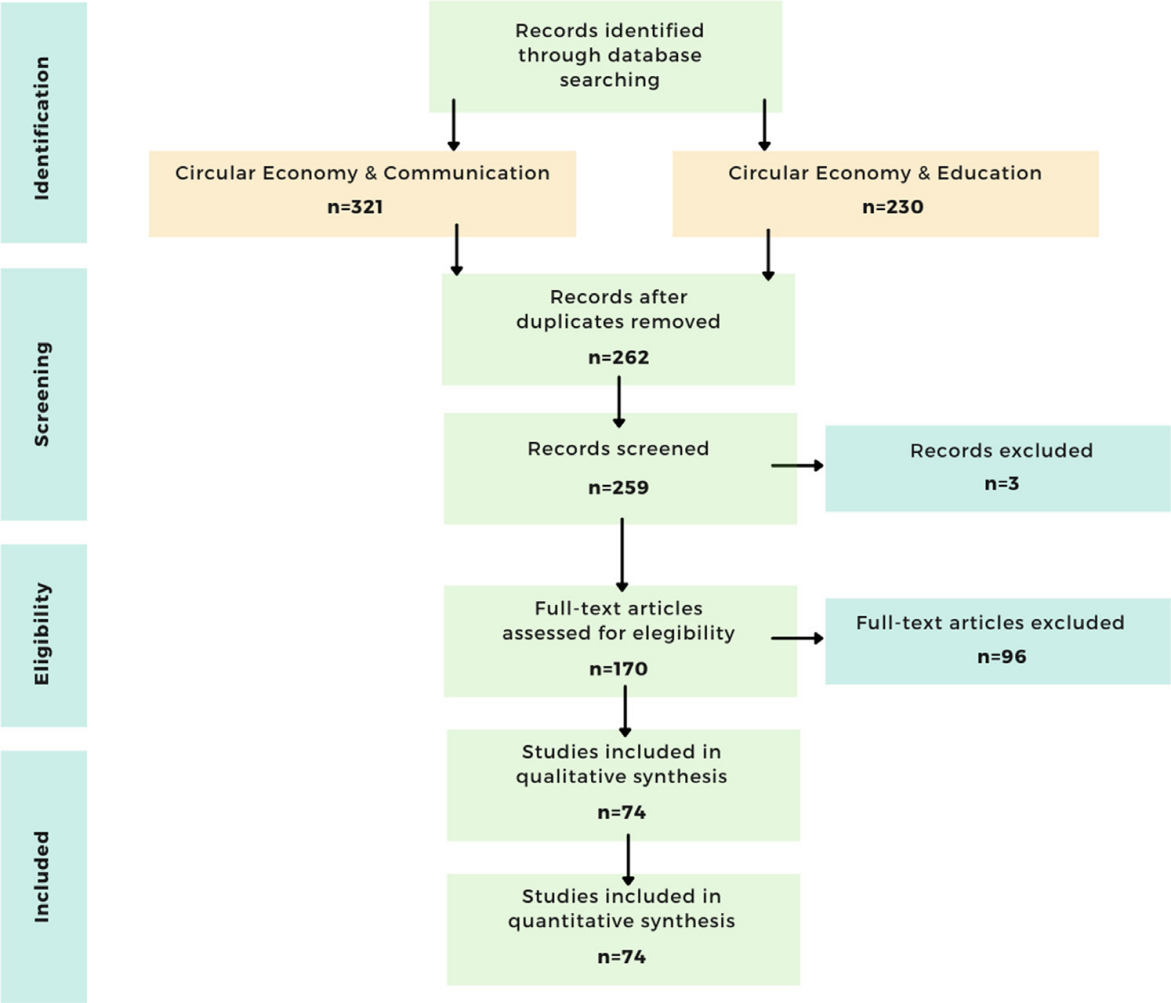
PRISMA statement flow diagram.

Once all publications were retrieved from the three databases, the four phases of the PRISMA statement (identification, screening, eligibility, and included) were followed to refine the selection process. Fig. 3 shows the PRISMA Statement Flow diagram.

The identification phase resulted in 551 publications (Articles, Proceedings papers, Editorial Material, and Reviews). Of these, 321 publications related to the circular economy AND communication, and 230 publications related to circular economy AND education. In the screening phase, duplicates were eliminated by unifying the records in a single document (n=262) (Articles and Proceedings papers). Then, records were manually checked, and three further duplicates were excluded. The publications’ citation metrics included in the data were current as of 2020. After evaluating the abstracts of the publications, those that were not related to the research area were excluded. Thus, 170 were determined eligible. These articles were read and reviewed in depth. After this review, 96 nonrelated publications and those that were not retrievable were excluded. Finally, 74 publications were included in the qualitative and quantitative analysis.

These 74 results were evaluated individually by applying the DESLOCIS Framework. Based on the analysis of the textual corpus of publications, a record file was completed with the different variables of analysis. Once the results were coded, the values are distributed by means of frequency tables.

Finally, a univariable and multivariable analysis was carried out, applying frequencies and vertical percentages.

To complete the multivariate analysis, the table of checks of proportions by column using the Z-test was used to determine any significant differences between the percentages compared [9].

## Ethics Statements

Before carrying out the research, the Ethics Committee of the Universidad Rey Juan Carlos approved its development and authorized it to be conducted (Authorization **ID 1806201910519**). The data do not require anonymity as they are scientific publications.

## CRediT authorship contribution statement

**Alejandro Carbonell-Alcocer:** Conceptualization, Methodology, Investigation, Data curation, Writing – original draft, Funding acquisition. **Juan Romero-Luis:** Conceptualization, Methodology, Investigation, Data curation, Writing – original draft, Funding acquisition. **Manuel Gertrudix:** Conceptualization, Methodology, Supervision, Writing – review & editing, Project administration, Funding acquisition. **Daniel Wuebben:** Conceptualization, Writing – review & editing.

## Declaration of Competing Interest

The authors declare that they have no known competing financial interests or personal relationships that could have appeared to influence the work reported in this paper.

## Data Availability

Sample Records. A systematic review in Circular Economy and Bioenergy addressed by Education and Communication (Original data) (Zenodo. OpenAIRE). Systematic Literature Review results: PRISMA Statement Phases for Education and Communication based on Circular Economy and Bioenergy Literature (Original data) (Zenodo. OpenAIRE). Descriptors for a systematic literature review on social sciences (DESLOCIS) (Original data) (Zenodo. OpenAIRE).
